# Changes in the physicochemical components, polyphenol profile, and flavor of persimmon wine during spontaneous and inoculated fermentation

**DOI:** 10.1002/fsn3.1560

**Published:** 2020-04-14

**Authors:** Yao Lu, Yaqiong Liu, Jiawei Lv, Yanli Ma, Xiaolei Guan

**Affiliations:** ^1^ College of Food Science and Technology Hebei Agricultural University Baoding Hebei China; ^2^ Guangxi talent highland of preservation and deep processing research in fruit and vegetables Hezhou University Hezhou Guangxi China; ^3^ Henan Key Laboratory of Industrial Microbial Resources and Fermentation Technology Nanyang Institute of Technology Nanyang China

**Keywords:** fermentation, flavor, sensory, oenological parameter, Persimmon wine, polyphenol

## Abstract

Changes in the oenological parameters, total phenols, total flavonoids, and individual phenols of persimmon during spontaneous and inoculated fermentation were investigated. The volatile compounds and sensory character of the persimmon wine were compared and evaluated simultaneously. Results show that at the end of fermentation, spontaneous persimmon wine (SPW) has higher contents of total flavonoids, total phenols yet lower concentrations of alcohol and volatile compounds than inoculated persimmon wine (IPW). Catechin, salicylic acid, quercetin, and vanillic acid were the main phenolic compounds in both types of persimmon wine. There are six volatile components in the IPW with an OAV greater than 1, which are isoamyl acetate, ethyl hexanoate, methyl octanoate, ethyl octanoate, phenethyl acetate, and 2, 4‐di‐tert‐butylphenol, and these compounds contribute to the IPW with brandy and fruity sensory properties, while only three volatile components in SPW have OAV greater than 1, which are isoamyl acetate, ethyl hexanoate, and ethyl octanoate. Spontaneous fermentation increased the proportion of esters and alcohols in the overall volatile compounds. During sensory evaluation, IPW was characterized by “brandy,” “bitterness,” and low “sweetness,” and SPW has a high score of “sweetness,” “balance,” desirable “color,” and “body.”

## Graphical abstract

Changes in the oenological parameters, total phenols, total flavonoids, and individual phenolic compounds of persimmon wine during spontaneous and inoculated fermentation were investigated. The volatile compounds and sensory character of the persimmon wines were compared and evaluated simultaneously.

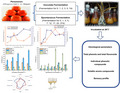

## 1 INTRODUCTION

China is the largest persimmon producer in the world, with crop area and yield of 981,528 ha and 4,216,376 t in 2017, accounting for 91.32% and 73.32% of the world's total area and production, respectively [FAOSTAT]. More than 1,000 original varieties of persimmon are known in China, 9 of which are completely sweet persimmon resources, whereas the rest are astringent persimmons (Li, Y, & Wang, R.Z., 2006). “Mopan” cultivar is a famous astringent persimmon variety with large fruit size and excellent sensory quality. This cultivar also has the largest growing areas and the highest yield in Northern China. However, only less than 10% of persimmon is processed into dried persimmons and other products, and the trade volume accounts for only approximately 3% of the global total yield (Liu, Zhu, & Li, 2016). A large fraction of persimmons is discarded every harvest period because of low price and lack of effective processing technology.

At present, the production of fruit wines is considered an attractive means of utilizing surplus and overripe fruits, decreasing losses, and adding value to raw material. Chinese fruit wine production increased by an annual rate of 15% from 2013 to 2017, especially in 2017, with an output of 2,330,900 kl (Zhao et al., 2019). In comparison with other fruits, such as apple, pear, and orange, persimmon has higher sugar content that comprises sucrose and its monomers (e.g., glucose and fructose), which provide abundant carbon sources for brewing (Itamura, Zheng, & Akaura, 2005). Moreover, persimmon is rich in polyphenols, carotenoids, total flavonoids, and tannin (Butt et al., 2015). These compounds are released into the aqueous ethanolic solution through the winemaking process, thereby improving their absorption and bioavailability during consumption (Shahidi, 2009). These bioactive compounds play a protective role on diabetes (Yaqub et al., 2016), hypercholesterolemia (Gorinstein et al., 2011), cancer (Direito et al., 2017), hypertension (Xie, Xie, Xu, & Yang, 2015), and Alzheimer's disease (Huang et al., 2016).

Processing methods and quality improvement of persimmon wine have been paid more attention and extensively investigated recently. Gorinstein et al. (1993) reported the effect of three different processing methods on the composition and sensory properties of persimmon liqueurs. In addition, the physicochemical and sensory characteristics of persimmon wine were influenced by the addition of nitrogen source and citric acid (Sharma, Mahant, Sharma, & Thakur, 2017). The persimmon wine at 25°C has high contents of total tannins, total flavonoids, and antioxidant activity (Liu et al., 2018). Aroma is an important factor that affects the sensory properties and acceptability of fruit wine. Odor activity value (OAV) is generally used to identify the contribution of volatile compounds to aroma (Rincon et al., 2019; Vilanova et al., 2012). OAV > 1 is deemed to contribute to wine aroma (Anon et al., 2014; Vilanova et al., 2012), and OAV > 0.2 may contribute to the aroma of wine through synergy (Meilgaard, 2001). Microorganisms play an important role in determining wine quality. Cofermentation can improve the content of aroma and is an ideal approach to produce persimmon wine (Liu, Bai, Shen, & Yu, 2012). Several works have shown that spontaneous fermentation positively affects wine quality because of the growth of different species and/or strains (Francesca et al., 2016; Maturano et al., 2019; Xu, Liu, Wang, & Kong, 2020). However, few studies have been conducted on the effects of spontaneous fermentation on the quality of persimmon wine.

This work was performed to evaluate the oenological parameters, total phenols, total flavonoids, individual phenolic, volatile compounds, and sensory characteristics during persimmon spontaneous and inoculated alcoholic fermentation. This study will help provide a deeper insight into the factors that affect the quality of persimmon wine and improve product quality.

## 2 MATERIAL AND METHODS

### 2.1 Materials

Persimmon (*Diospyros kaki* L. cv. Mopan) was collected from Baoding City, Hebei Province of China in October 2018. The fruits were treated with CO_2_ to remove astringency and stored at −20°C until use. Active wine dry yeast (*Saccharomyces cerevisiae*) was purchased from Angel Yeast Co., Ltd. (Yichang, China).

The standard compounds of gallic acid, protocatechuic acid, catechin, vanillic acid, syringic acid, 4‐coumaric acid, syringaldehyde, ferulic acid, guaiacol, benzoic acid, salicylic acid, quercetin, 3‐octanol and methanol, ethanol, acetonitrile (HPLC grade), acetic acid, rutin, and ethyl acetate were purchased from Sigma‐Aldrich (St. Louis). Folin–Ciocalteu, Na_2_CO_3_, Al (NO)_3_, Na OH, HCl, Fehling reagent, glucose, pectinase (pectinase > 3.5 × 10^4^ U g^−1^, cellulase > 9 × 10^4^ U g^− 1^), and other chemicals were of analytical grade and purchased from Fluka (Buchs, Switzerland).

### 2.2 Persimmon wine

Vinification was conducted according to previous reports (Zhu et al., 2016). After thawing 15 kg of persimmons and removing the calyx, it was crushed and enzymatically hydrolysed with pectinase (1 g/kg, slurry) at 30 ± 1°C for 2 hr. Then, citric acid was added to the pulp to adjust the pH to 3.7. The initial soluble solid content of the pulp was determined to be 18.2 ± 1.0° Brix. The persimmon pulp was distributed into thirteen 500‐mL volume glass bottles with a working volume of 350 ml. *S. cerevisiae* strain (0.2 g/kg, slurry) was added to five bottles for inoculated fermentation. Subsequently, the cultures were incubated at 28°C.

Samples were taken 6 times during the inoculated fermentation at days 0, 1, 2, 3, 5, and 7 of fermentation and taken 9 times during spontaneous fermentation on days 0, 1, 3, 5, 7, 12, 17, 22, and 27. Then, fermented persimmon slurry was centrifuged (4,700× *g*, 10 min) and the supernatant was stored at −20°C until analysis. All experiments were elaborated in triplicate yielding 45 samples.

### 2.3 Oenological parameters

The reducing sugar was determined according to Xiong, Li, Xie, Xue, and Sun (2014). The titratable acidity (expressed as g/L tartaric acid) was determined using AOAC Official Method 962.12. Methanol and ethanol were determined using an Agilent Model 7890B gas chromatograph (GC) with reference to the national standard method GB 5009.266–2016 and GB 5009.225–2016 (SAC, 2016) published by the Standardization Administration of China (SAC).

### 2.4 Determination of total phenols, total flavonoids, and individual phenols

The total phenols were quantified by the Folin–Ciocalteu method. After centrifugation at 4,700 *g* for 10 min, 1 ml of the supernatant was taken and diluted in the absorbance of 0.2 and 0.8. The diluent (0.5 ml) was successively mixed with distilled water (2.5 ml), Folin–Ciocalteu reagent (0.5 ml), and 7.5% (w/v) Na_2_CO_3_ (1.5 ml). The mixture was reacted in the dark at room temperature for 2 hr, and the absorbance was read at a wavelength of 765 nm by the spectrophotometer. The total phenols of test samples were presented as per mg of gallic acid equivalents per 1 L of persimmon pulp. The total phenol content during the fermentation of persimmon was calculated according to the following gallic acid calibration curve: y = 0.0651 x + 0.002 (*R* ^2^ = 0.9996).

The total flavonoids were determined according to Xu et al. (2019) and with some modifications. The above supernatant (1 ml) was put in a 10‐mL volumetric flask and mixed with 5% (w/v) NaNO_2_ (0.5 ml). After 6 min, 10% (w/v) Al (NO)_3_ (0.5 ml) was added. After another 6 min, 4% NaOH (4 ml) was added and distilled water was added after 15 min up to the volume tick mark. Absorbance was read at 510 nm by the spectrophotometer, and the results were reported as milligram per liter of rutin equivalents (mg RT/L). The content of total flavonoids during the fermentation of persimmon was calculated according to the following rutin calibration curve: *y* = 0.004 *x* + 0.0061 (*R* ^2^ = 0.9989).

The individual phenols were determined by high‐performance liquid chromatography (HPLC). The samples were prepared using method of Ye, Yue, and Yuan (2014). HPLC measurement conditions refer to the method of Xia and with modification (Wang et al., 2017). Chromatographic conditions were as follows: the mobile phase, water–acetic acid (98:2, v/v) (A), and acetonitrile (B); column, Waters X‐TerraMS C18 column (250 mm × 4.0 mm, 5.0 μm particle size); detection wavelength, 280 nm; injection volume, 10 μl; flow rate, 1 ml/min; column temperature, 30°C; elution program; and 0–5 min of 3% B, 5–15 min of 3%–10% B, 15–25 min of 15%–25% B, 25–35 min of 25%–30% B, 35–40 min of 30%–3% B, and 40–42 min of 3% B. External standard was used for quantitative analysis. The experiment was carried out in triplicate.

### 2.5 Determination of volatile compounds

HS‐SPME (50/30 µm DVB/CAR/PDMS, Supelco USA) was used to extract the volatile compounds in the inoculated persimmon wine (IPW) and spontaneous persimmon wine (SPW). GC coupled with MS (GC‐MS, 7890B– 5977A, Agilent, USA) was used for separation and identification. A volume of 25 µl of the internal standard (3‐octanol, 300 mg/ml), 1g sodium chloride, and 7.5 µml of the persimmon fermentation were added to a 20‐ml headspace vial. The sample was statically incubated at 40°C for 15 min, followed by a 45‐min extraction of the volatile compounds by an SPME fiber.

The GC conditions were set as follows: Column, HP‐Innowax (60 m × 0.25 mm × 0.25 µm); carrier gas (e.g. He) velocity, 1.4 ml/min; injection temperature, 240°C; splitless mode; oven temperature program at 50°C (maintained 2 min) to 80°C by 3°C/min, then raised to 230°C by 5°C/min; and the final temperature stage retained for 6 min. The MS conditions were set as follows: electron impact (EI) mode at 70 eV, temperature of 230°C, and total ion current scanning range of 33–550 m/z.

#### 2.5.1 Qualitative analysis

The NIST 14 library was used for comparison, the internal standard method was used for quantification, and the components with a matching degree of more than 80% were analyzed. For each sample, triplicate extractions were performed and used for analyses.

### 2.6 Sensory evaluation

Eight trained panellists (3 males and 5 females) used descriptive analysis to describe the sensory profile of the IPW and SPW. Aliquots of 20 ml of the wines at 20°C were poured into fruit wine tasting glasses, encoded with three random numbers, and presented in random order. The panellists defined the sensory attributes as color, transparency, persimmon, brandy, acidity, sweetness, astringency, bitterness, body, and balance. The wine samples were analyzed with a nonstructured scale of 10 cm, in which a 0 score indicated “not perceptible” and a score of 10 indicated “strongly perceptible.” The results are expressed as average values.

### 2.7 Statistical analysis

All data were analyzed using SPSS 23.0 with Duncan, and the level of significance was set to *p* < .05. The reported results are mean values ± standard deviation of triplicates. Sensory evaluation was represented by a spider web pattern.

## 3 RESULTS AND DISCUSSION

### 3.1 Evolution of oenological parameters during fermentation

Changes in the oenological parameters, such as reducing sugar, titratable acid, ethanol, and methanol, during fermentation are listed in Table 1. During the first 3 d of the inoculated fermentation period, the amount of reducing sugar decreased substantially from 19.18 ± 0.02 g/L to 1.18 ± 0.03 g/L and slightly changed thereafter. At the end of the fermentation, the content decreased to 0.93 ± 0.01 g/L, thereby agreeing with the result in Liu et al. (2018). By contrast, in the spontaneous fermentation, the reducing sugar content declined apparently in the first 7 d and the last 5 d until 7.63 ± 0.37 g/L. The ethanol concentration increased with prolonged fermentation time, reaching 7.86 ± 0.31% *v/v* and 4.62 ± 0.96% *v/v* at the end of inoculated and spontaneous fermentations, respectively. This phenomenon was consistent with the results of Ciani et al studies that mixed fermentation of Saccharomyces cerevisiae and non‐Saccharomyces cerevisiae increases the total acid content of the wine and reduces the ethanol content, which may be due to the different yeast species and/or strains producing toxic compounds or compete for nutrition and participate in multiple interactions (Ciani, Comitini, Mannazzu, & Domizio, 2010). In particular, the production of medium‐chain fatty acids and large amounts of acetic acid can adversely affect the growth of cofermented yeast material (Cinai & Comitini, 2015). But it is slightly different from other studies (Liu, Yang, Qi, Fan, & Wei, 2017; Zhu et al., 2016).

**Table 1:** Changes in basic physicochemical parameters during persimmon wine inoculated and spontaneous fermentation (*n* = 3)

Fermentation	Time (d)	Parameters			
		Reducing sugar(g/L)	Titratable acid (g/L)	Ethanol (% v/v)	Methanol (g/L)
Inoculation	0	19.18 ± 0.02^a^	2.40 ± 0.02^d^	ND	ND
	1	9.56 ± 0.5^b^	3.53 ± 0.01^c^	3.02 ± 1.02^d^	0.34 ± 0.07^a^
	2	3.13 ± 0.34^c^	3.40 ± 0.32^c^	4.77 ± 0.15^c^	0.21 ± 0.03^b^
	3	1.18 ± 0.03^d^	4.29 ± 0.09^b^	6.65 ± 0.29^b^	0.16 ± 0.03^b^
	5	1.05 ± 0.08^d^	5.23 ± 0.09^ab^	6.46 ± 0.13^b^	0.14 ± 0.04^b^
	7	0.93 ± 0.01^Bd^	5.40 ± 0.11^Aa^	7.86 ± 0.31^Aa^	0.13 ± 0.03^Bb^
Spontaneous	0	21.93 ± 0.13^b^	2.70 ± 0.00^b^	ND	ND
	1	27.22 ± 0.80^a^	2.82 ± 0.78^b^	1.68 ± 0.01^h^	0.60 ± 0.20^a^
	3	18.27 ± 1.94^c^	3.33 ± 0.48^b^	1.79 ± 0.15^g^	0.59 ± 0.17^ab^
	5	15.38 ± 1.46^cd^	3.18 ± 0.61^b^	1.96 ± 0.20^f^	0.52 ± 0.07^ab^
	7	15.43 ± 0.65^cd^	4.32 ± 0.26^b^	2.11 ± 0.20^e^	0.39 ± 0.08^bc^
	12	13.83 ± 2.90^d^	9.04 ± 3.97^a^	2.41 ± 0.37^d^	0.40 ± 0.03^bc^
	17	13.29 ± 1.25^d^	7.23 ± 1.31^ab^	3.17 ± 0.12^b^	0.43 ± 0.04^abc^
	22	12.75 ± 2.07^d^	6.84 ± 4.36^ab^	2.72 ± 0.93^c^	0.24 ± 0.04^c^
	27	7.63 ± 0.37^Ae^	4.45 ± 2.09^Ab^	4.62 ± 0.96^Ba^	0.26 ± 0.03^Ac^

Note:

Different letters (A, B) indicated significant differences among the sample of 7d (inoculation) and 27d (spontaneous) in the columns (*p* < .05), and the same letter means no significant difference(*p* > .05). Values with different letters in the same column (a–e) are significantly different (*p* < .05) from each other.

Abbreviation: *ND*, not detected.

The total titratable acid increased steadily from 2.40 ± 0.02 g/L to 5.40 ± 0.11 g/L during inoculated fermentation. It increased until the first 12 d and then dropped during spontaneous fermentation. The concentration of methanol, as a harmful substance produced in winemaking, should not exceed 0.4 g/L, as stipulated by GB/T 15037–2008 (SAC, 2006) of China. Methanol content decreased during the fermentation, and the contents of IPW and SPW were 0.13 ± 0.03g/L and 0.26 ± 0.03g/L, respectively, which met the national criteria.

### 3.2 Evolution of total phenols, total flavonoids, and individual phenols

**Figure 1:**
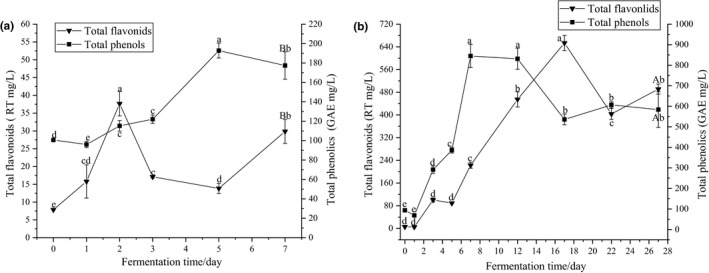
Changes in total flavonoids and total phenols in inoculated (a) and spontaneous (b) persimmon wine fermentation. Different letters indicated significant differences according to Duncan's test (*p* < .05)

Figure 1 shows the variations in the total phenols and flavonoids during fermentation. As fermentation proceeded, the total phenols first increased and then decreased. The content of the total phenols reached a peak on the 5th day (192.78 ± 7.60 mg/L) of inoculated and on the 7th day (844.85 ± 56.48 mg/L) of spontaneous fermentation. The SPW showed higher content of total phenols than IPW, with content reaching 583.87 ± 86.68 mg/L and 177.42 ± 14.12 mg/L. Furthermore, the total phenols’ concentration in the SPW was lower than the published value in Liu et al., 2018. This difference may be related not only to the persimmon variety and fermentation technology but also to the microbial communities in the persimmon pulp. The change in the total flavonoids was similar to the total phenols. The content of the total flavonoids reached its peak (37.67 ± 3.45 mg/L) on the second day of the inoculated fermentation and maximum value (623.50 ± 83.06 mg/L) on the 17th day under spontaneous fermentation. The total flavonoid content was significantly higher in the SPW (488.48 ± 21.24 mg/L) than in IPW (29.85 ± 3.36 mg/L). Some phenols were transferred from persimmon fruits into the wine during alcoholic fermentation. The leaching rate of the total phenols and total flavonoids increased with the ethanol concentration at the initial stage of fermentation. The subsequent decrease in phenols may be caused by self‐oxidation or reaction with other substances (Zhang, Chang, Stringer, & Zhang, 2017). In addition, the concentration of phenols was related to enzymatic reactions or metabolic activities of yeasts during alcoholic fermentation (Ribéreau‐Gayon, Glories, Maujean, & Bourdieu, 2006). Minnaar et al reported that coinoculation fermentations (*S. cerevisiae* strains and non‐*Saccharomyces* yeasts and lactic acid) increased phenolics in Syrah wines. There were also found that spontaneous fermentations and cofermentations with *Hanseniaspora* vineae improved the sensory and quality of Chardonnay wines by increasing phenolic concentrations (Medina et al., 2013). Therefore, the significant difference in the content of phenols between IPW and SPW may be due to the different composition of microbial community in the fermentation process, but further research was needed.

**Figure 2:**
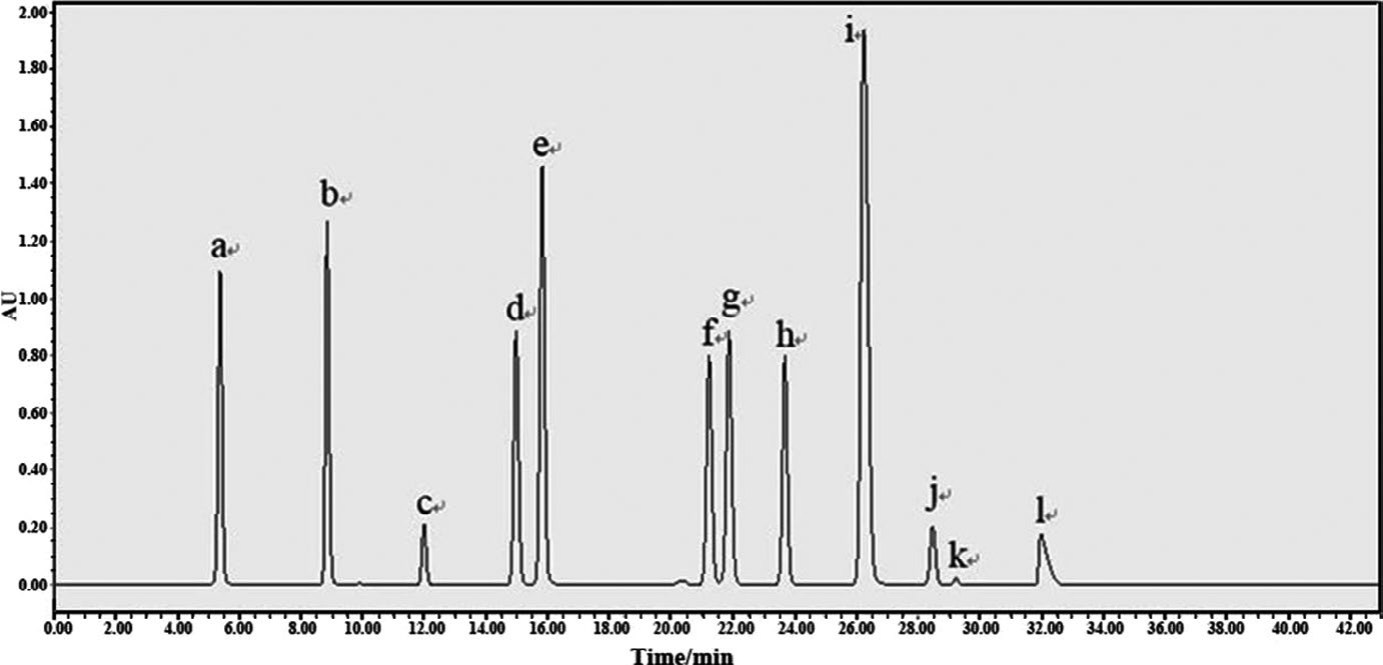
HPLC chromatogram of 12 monophenols of inoculated and spontaneous persimmon wine fermentation detected at 280 nm. a—Gallic acid, b—Protocatechuic acid, c—Catechin, d—Vanillic acid, e—Syringic acid, f—4‐Coumaric acid, g—Syringaldehyde, h—Ferulic acid, i—Guaiacol, j—Benzoic acid, k—Salicylic acid, l—Quercetin [Correction added on 5 May 2020, after first online publication: Figure 2 has been corrected.]

Figure 2 shows the chromatogram of 12 monomer phenols identified and quantified by HPLC analysis during the fermentation process. Changes in the monomer phenol content during fermentation are shown in Tables 2 and 3. As can be seen from the table, the content of individual phenolics initially increased and then decreased during the inoculated fermentation. By contrast, spontaneous fermentation showed a more complex variation. Catechin showed the highest component in the IPW and SPW, accounting for 65.14% and 32.03% of the total contents, respectively, followed by salicylic acid, quercetin, and vanillic acid. Contrary to other studies, gallic acid was relatively low in this experiment. The fermentation method affected the content of catechin, salicylic acid, quercetin, vanillic acid, benzoic acid, and syringic acid. Compared with IPW, the SPW showed higher contents of these phenolics at corresponding values of 478.5 ± 3.5, 84.0 ± 12.7, 82.5 ± 4.9, 47.5 ± 2.1, 15.5 ± 0.7, and 6.0 ± 1.4 mg/L. These values were higher than the results of Guo, Li, Wang, Guo, and Wu (2017). At the end of the fermentation, the contents of the other 11 monomeric phenols, except for ferulic acid, were higher than those in the persimmon mush. This result suggested that alcoholic fermentation increased the content of phenolic substances, in accordance with existing data (Zou, Wu, Yu, Xiao, & Xu, 2017). Given the different climatic conditions, harvest time, processing, available nutrients, and other factors, the phenol content differs greatly among varieties of persimmon (Tchabo et al., 2017). Individual phenolics found in persimmon included (+)‐catechin, caffeic acid, chlorogenic acid, epigallocatechin, ferulic acid, gallic acid, o‐phthalic acid, *p*‐coumaric acid, phloridzin, *p*‐hydroxybenzoic acid, quercetin, rutin, syringic acid, and vanillic acid (Perez‐Burillo, Oliveras, Quesada, Rufian‐Henares, & Pastoriza, 2018). During fermentation, the compounds and concentration of phenolics were changed because of the action of the various enzymes (excreted during the metabolism of the yeast) on the conjugated phenolics to liberate the phenolics (Zhang et al., 2017).

**Table 2:** Changes in individual phenol content in inoculated persimmon wine fermentation process (*n* = 3)

Phenolic compounds (mg/L)	Fermentation time (d)					
	0	1	2	3	5	7
Gallic acid	7.5 ± 0.7^d^	6.5 ± 0.7^e^	2.5 ± 0.7^f^	20.5 ± 0.7^a^	11.7 ± 1.5^b^	9.5 ± 0.7^c^
Protocatechuic acid*	2.5 ± 0.8	7.5 ± 0.6	1.0 ± 0.0	6.0 ± 1.4	5.0 ± 1.0	3.5 ± 0.7
Catechin*	30.0 ± 8.4^f^	42.0 ± 8.0^e^	102.5 ± 12.1^c^	150.5 ± 11.8^b^	167.7 ± 44.1^a^	57.5 ± 5.0^d^
Vanillic acid*	0.5 ± 0.2^c^	1.0 ± 0.4^bc^	3.5 ± 0.7^b^	3.5 ± 0.7^b^	0.7 ± 0.6^c^	11.5 ± 2.1^a^
Syringic acid*	1.0 ± 0.0^bc^	1.5 ± 0.7^b^	ND	14.0 ± 1.4^a^	1.0 ± 0.0^bc^	1.0 ± 0.0^bc^
4‐Coumaric acid*	1.0 ± 0.2	0.5 ± 0.1	ND	1.0 ± 0.4	1.0 ± 0.0	1.0 ± 0.0
Syringaldehyde*	1.0 ± 0.0	1.0 ± 0.0	1.0 ± 0.0	0.5 ± 0.0	1.0 ± 0.0	1.0 ± 0.0
Ferulic acid*	3.5 ± 2.1^cd^	20.5 ± 3.5^a^	10.5 ± 0.7^bc^	14.5 ± 7.7^ab^	2.3 ± 0.6^d^	1.0 ± 0.0^d^
Guaiacol	ND	ND	ND	ND	ND	1.0 ± 0.0
Benzoic acid*	5.5 ± 0.1^a^	8.5 ± 2.1^b^	ND	5.5 ± 0.7^b^	8.0 ± 1.0^b^	7.5 ± 0.7^b^
Salicylic acid*	42.0 ± 3.1^c^	248 ± 29.4^b^	372.0 ± 9.9^a^	300.0 ± 42.3^b^	61.3 ± 6.1^cd^	52.0 ± 7.1^d^
Quercetin*	27.6 ± 1.1^b^	125.5 ± 2.1^a^	110.5 ± 0.7^a^	115.5 ± 0.7^a^	44.0 ± 4.6^bc^	34.0 ± 2.8^c^
∑	120.6 ± 11.1^e^	462.5 ± 47.2^c^	602.0 ± 24.7^b^	631.5 ± 67.8^a^	302.7 ± 59.5^d^	179.5 ± 19.1^f^

Note:

Values with different letters in the same line (a–f) are significantly different (*p* < .05) from each other. Letters are not reported if the four values are not significantly different

Abbreviation: ND, not detected

* Indicated significant differences among the sample of 7d (inoculation) and 27d (spontaneous) in the same individual phenols (*p* < .05).

**Table 3:** Changes in individual phenol content in spontaneous persimmon wine fermentation process (*n* = 3)

Phenolic compounds (mg/L)	Fermentation time (d)								
	0	1	3	5	7	12	17	22	27
Gallic acid	5.5 ± 0.7^b^	7.8 ± 1.7^b^	8.0 ± 1.4^b^	5.7 ± 0.6^b^	7.5 ± 0.7^b^	3.5 ± 0.7^b^	40.0 ± 7.2^a^	8.3 ± 0.6^b^	7.5 ± 2.1^b^
Protocatechuic acid*	3.0 ± 1.4^ef^	2.0 ± 0.8^f^	11.0 ± 1.4^b^	3.3 ± 0.6^def^	6.0 ± 1.4^cd^	15.5 ± 0.7^a^	6.0 ± 2.6^cd^	7. 7 ± 0.6^c^	5.5 ± 0.7^cde^
Catechin*	16.0 ± 4.2^f^	41.3 ± 2.2^e^	16.5 ± 0.5^f^	25.0 ± 1.0^ef^	76.5 ± 4.9^d^	122.0 ± 1.4^c^	184.0 ± 20.6^b^	23. 7 ± 0.6e^f^	478.5 ± 3.5^a^
Vanillic acid*	2.5 ± 0.7^fg^	14.3 ± 1.7^e^	0.5 ± 0.1^g^	4.3 ± 0.6^f^	3.5 ± 0.7^fg^	18.5 ± 2.1^d^	22.0 ± 1.0^c^	26.7 ± 3.1^b^	47.5 ± 2.1^a^
Syringic acid*	1.0 ± 0.0^ef^	3.0 ± 0.8d^e^	0.5 ± 0.0^f^	2.0 ± 1.0^def^	1.0 ± 0.0^ef^	2.5 ± 0.7^def^	15.0 ± 2.0^a^	3.7 ± 0.6^c^	6.0 ± 1.4^b^
4‐Coumaric acid*	1.0 ± 0.0^d^	11.5 ± 2.4^c^	0.5 ± 0.1^d^	7.7 ± 0.6^c^	1.5 ± 0.7^d^	16.5 ± 3.5^b^	65.3 ± 3.5^a^	3.3 ± 0.6^d^	3.5 ± 0.7^d^
Syringaldehyde*	1.0 ± 0.0^c^	1.0 ± 0.0^c^	1.0 ± 0.2^c^	1.0 ± 0.0^c^	1.0 ± 0.0^c^	2.0 ± 1.4^abc^	2.7 ± 0.6^ab^	3.0 ± 1.0^a^	1.5 ± 0.7b^c^
Ferulic acid*	7.5 ± 2.1^d^	4.5 ± 0.6^e^	12.0 ± 2.8^bc^	2.0 ± 1.0^ef^	10.0 ± 1.4^cd^	26.5 ± 2.1^a^	13.3 ± 0.6^b^	4.0 ± 1.0^ef^	1.5 ± 0.7^f^
Guaiacol	ND	4.3 ± 1.0^a^	ND	0.7 ± 0.6^b^	ND	ND	4.3 ± 1.5^a^	0.7 ± 0.6^b^	1.0 ± 0.0^b^
Benzoic acid*	6.5 ± 0.7^cd^	4.5 ± 0.6^de^	4.5 ± 0.7^de^	4.0 ± 1.0^de^	5.5 ± 0.7^de^	3.0 ± 1.4^e^	12.0 ± 1.0^b^	8.3 ± 2.5^c^	15.5 ± 0.7^a^
Salicylic acid*	57.0 ± 4.2^c^	36.5 ± 11.9^d^	309.0 ± 9.9^a^	4.7 ± 1.5^e^	8.5 ± 0.7^e^	16.0 ± 2.8^e^	56.3 ± 7.6^c^	34.3 ± 10.3^d^	84.0 ± 12.7^b^
Quercetin*	29.0 ± 4.2^f^	32.3 ± 8.5^f^	107.5 ± 2.1^c^	26.0 ± 1.0^f^	124.5 ± 6.4^b^	291.5 ± 6.4^a^	44. 3 ± 0.6^e^	32. 3 ± 2.5^f^	82.5 ± 4.9^d^
∑	130.0 ± 17.3^h^	163.0 ± 28.4^f^	471.0 ± 15.2^c^	86.4 ± 5.6^i^	245.5 ± 16.3^e^	517.0 ± 28.8^b^	465.2 ± 45.7^d^	155.7 ± 22.7^g^	734.5 ± 27.4^a^

Note:

Values with different letters in the same line (a–f) are significantly different (*p* < .05) from each other

ND, not detected.

* Indicated significant differences among the sample of 7d (inoculation) and 27d (spontaneous) in the same individual phenols (*p* < .05).

### 3.3 Volatile compounds

Table 4 shows the volatile aroma components in IPW and SPW. The detected components were divided into esters, alcohols, acids, and others. There are 25 types of volatile components in IPW and 20 types in SPW. Spontaneous fermentation reduced the type and content of esters and alcohols, which may be related to the higher content of individual phenolics. The interactions of π–π stacking of the galloyl and aromatic rings of the odor molecules, such as gallic acid and catechin, may reduce the volatility of aroma, and the effect of phenolic acids on aroma volatility has been associated with the matrix composition of the wine (Jung & Ebeler, 2003; Perez‐Jimenez, Chaya, & Pozo‐Bayon, 2019).

**Table 4:** Volatile aroma compounds of IPW and SPW. All data are expressed as means ± *SD* of three analyses

Category	Compounds	Odor threshold (μg/L)	IPW		SPW	
			Contents (μg/L)	OAV	Contents (μg/L)	OAV
Ester (10)	Isoamyl acetate	30^A^	201.2 ± 27.80	6.71	197.0 ± 9.90	6.57
	Ethyl hexanoate	14^B^	166.1 ± 38.90^a^	11.86	84.7 ± 5.98^b^	6.05
	Methyl octanoate	200^E^	331.5 ± 42.18	1.66	ND	
	Ethyl octanoate	5^D^	874.3 ± 0.38^a^	174.8	133.2 ± 5.43^b^	26.64
	Phenethyl acetate	250^A^	279.7 ± 17.00^a^	1.12	32.2 ± 1.3^b^	0.13
	Ethyl caprate	200^A^	166.8 ± 2.64^a^	0.83	25.7 ± 0.00^b^	0.13
	Ethyl benzoate		ND		12.8 ± 0.95	
	Ethyl 9‐hexadecenoate		ND		1.9 ± 0.00	
	Ethyl palmitate	1500^D^	ND		2.7 ± 0.02	0.00
	Ethyl laurate	500^E^	6.6 ± 0.40	0.02	ND	
	∑		1575.5 ± 30.02^a^		477.4 ± 22.2^b^	
Alcohols (9)	Hexyl alcohol	8000^A^	48.6 ± 2.30^a^	0.93	25.3 ± 6.80^b^	0.00
	Terpinen−4‐ol		ND		16.0 ± 1.50	
	2‐Methyl−1‐propanol	30000^B^	72.8 ± 1.70		ND	
	Furfuryl alcohol		8.4 ± 1.90		ND	
	3‐Methyl−1‐butanol	30000^E^	2,995.5 ± 143.90	0.10	ND	
	2,3‐Butanediol	120000^F^	90.2 ± 4.54^a^		10.9 ± 1.15^b^	
	3‐methyl−1‐pentanol		8.6 ± 0.00		ND	
	3‐Methylthiopropanol		5.7 ± 0.01		ND	
	Phenethyl alcohol	14000^C^	1803.8 ± 9.40^a^	0.13	442.1 ± 3.21^b^	0.03
	∑		5,026.4 ± 16.73^a^		494.3 ± 26.00^b^	
Acids (5)	Acetic acid glacial	200000^B^	485.3 ± 18.69	0.002	481.2 ± 55.89	0.002
	Hexanoic acid	420^B^	62.2 ± 3.80^a^	0.15	10.8 ± 0.1^b^	0.03
	Octanoic acid	500^B^	227.4 ± 17.30^a^	0.45	17.5 ± 1.15^b^	0.04
	Decanoic acid	1000^B^	130.4 ± 7.70^a^	0.13	7.3 ± 0.38^b^	0.01
	Lauric acid		6.3 ± 0.04^c^		ND	
	∑		911.6 ± 16.53^a^		511.4 ± 53.59^b^	
Others (5)	2,4‐Di‐tert‐butylphenol	200^F^	324.8 ± 10.56^a^	1.62	21.8 ± 0.13^b^	0.11
	Styrol		27.5 ± 1.17^b^		67.7 ± 2.78^a^	
	Naphthalene		6.8 ± 3.1		2.2 ± 0.04	
	Phenylacetaldehyde		23.8 ± 5.5		ND	
	Benzaldehyde	2000^D^	6.4 ± 0.04^a^	0.0	3.2 ± 0.02^b^	0.00
	∑		226.9 ± 20.91^a^		95.0 ± 2.88^b^	

Abbreviations: IPW, inoculated fermentation persimmon wine; *ND*, not detected; SPW, spontaneous fermentation persimmon wine.

^a,b^—different letters indicated significant differences among the sample of IPW and SPW in the line (*p* < .05), and letters are not reported if the four values are not significantly different. (*p* > .05).

^A^Guth (1997), ^B^Ferreira, López, and Cacho (2000), ^C^Zhao, Qian, He, Li, and Qian (2017), ^D^Etiévant (1991)， ^E^Pino and Queris (2010), ^F^Moyano, Zea, Moreno, and Medina (2002).

Esters and alcohols, as important aroma substances, have important effects on the flavor of the wine body. Seven kinds of esters were identified in the IPW, accounting for 20% of the total, among which the content of ethyl octanoate was the highest at 874.3 ± 0.38 μg/L, followed by ethyl hexanoate, isoamyl acetate, methyl octanoate, and phenethyl acetate. The concentrations of these compounds were above their threshold values (OAV > 1), indicating that they have a significant contribution to the aroma of persimmon wine. The contents of ethyl octanoate and ethyl hexanoate were 10 times higher than that of their threshold values (OAV > 10). These esters contribute to the persimmon wine with desirable fruity and brandy sensory properties, including pineapple, banana, apple, and strawberry (Kong et al., 2019). In SPW, eight esters were identified, accounting for approximately 30% of the total components, of which isoamyl acetate content was the highest (197.0 ± 9.90 μg/L). Isoamyl acetate presented OAV > 1, ethyl octanoate presented OAV > 10. Ethyl benzoate, ethyl 9‐hexadecenoate, and ethyl palmitate were only found in the SPW. However, the spontaneous fermentation employed in this study showed decrease in volatile esters in comparison with inoculated fermentation. This was consistent with the results of Li et al (Li et al., 2020), which was speculated that ester synthesis ability of yeasts was restricted, may be due to the growth inhibition caused by wild yeasts or other microorganisms during alcoholic fermentation.

Eight kinds of alcohols, accounting for 53% of the total components, with the highest alcohol as 3‐methyl‐1‐butanol (2,995.5 ± 143.90 μg/L), followed by phenethyl alcohol (1803.8 ± 9.40 μg/L), 2, 3‐butanediol (90.2 ± 4.54 μg/L), and 2‐methyl‐1‐propanol (72.8 ± 1.70 μg/L) were identified in the IPW. Four kinds of alcohols, accounting for 28% of the total contents, were identified in the SPW. The concentration of phenethyl alcohol reached 442.1 ± 3.21 μg/L, which was the highest among the alcohols. However, it presented an OAV < 0.1 because of the high threshold. Thus, this type of alcohol did not contribute to the overall flavor. It is noted that spontaneous fermentation was associated with a significantly lower production of many alcohols, especially in the case of 3‐methyl‐1‐butanol, 2, 3‐butanediol, and 2‐methyl‐1‐propanol compared with IPW. In addition, the content of ester compounds in SPW was lower than that in IPW, which suggested that the less content of alcohols might be the reason for the low content of related ethyl esters, such as ethyl hexanoate and ethyl caprate. The diverse types and contents of esters and alcohols in the IPW and SPW may have been caused by the metabolic discrepancy due to different microbial environment (De Filippis et al., 2019; Torrens et al., 2008).

Acids accounted for approximately 10% of the total volatile aroma in IPW, which contains acetic acid, glacial acid, and octanoic acid as the main acid compounds. Octanoic acid presented an OAV > 0.2, indicating its contribution to the overall aroma. Volatile acids in the SPW accounted for approximately 32% of the total components, the highest content of which is acetic acid glacial (481.2 ± 55.89 μg/L). Other components accounted for 2.9% and 8.6% of the total aroma in the IPW and SPW, respectively, among which 2, 4‐di‐tert‐butylphenol in IPW presented an OAV > 1.

### 3.4 Sensory analysis

**Figure 3:**
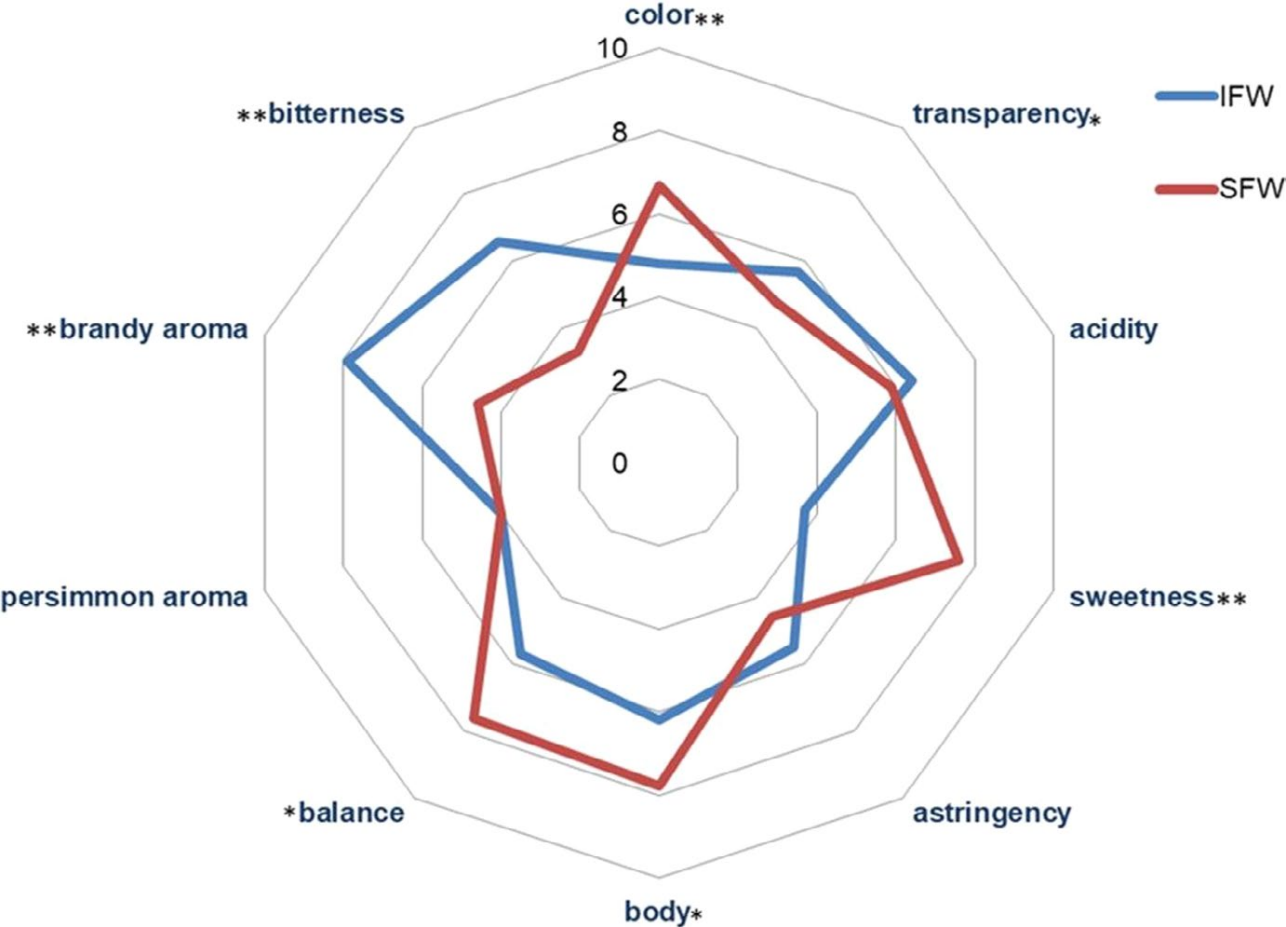
Spider plot for average values of sensory evaluation scores of IPW and SPW using 0–10 scale. The significances of differences between IPW and SPW were, respectively, *p < .05, **p < 0.01 and *p* > .05. IPW, inoculated fermentation persimmon wine, SPW, spontaneous fermentation persimmon wine [Correction added on 5 May 2020, after first online publication: Figure 3 has been corrected.]

Figure 3 illustrates the sensory evaluation of IPW and SPW. Analysis of variance showed significant differences (*p* < .05) for seven aroma descriptors. The IPW had significantly increased characteristics of brandy aroma, bitterness, and transparency and a decreased sweetness, body, and balance, compared with the SPW. The color of SPW was closer to the yellow of persimmon itself, while IPW was colorless. In terms of persimmon aroma, astringency, and acidity, there was no significant difference between the two finished persimmon wines despite the different scores. Overall, sensory evaluation results were consistent with oenological parameters (sugar content and ethanol content) and the content of volatile compounds in the IPW and SPW. IPW was distinguished by its reinforced “brandy aroma,” “bitterness,” and low “sweetness” descriptors, and this might be because ethyl octanoate and 3‐methyl‐1‐butanol were present in high concentrations. With regard to SPW, it was found “sweetness,” “balance,” and “body” received the high ratings.

## 4 CONCLUSIONS

Comparing the two kinds of alcoholic fermentation, the spontaneous fermentation increased the content of total flavonoids and phenols of persimmon wines, but reduced the content of ethanol. Catechin, salicylic acid, quercetin, and vanillic acid are the main individual phenol species in IPW and SPW. These results suggest that spontaneous fermentation may be improved the health‐promoting qualities of persimmon wine. Inoculated fermentation produced higher levels of volatile aromatic compounds than spontaneous fermentation but lower alcohols and esters‐to‐total aroma ratio than spontaneous fermentation. So the SPW has the more balance and soft body but less brandy aroma and bitterness sensory characteristic than IPW. Therefore, it is of great significance to isolate yeast (*Saccharomyces cerevisiae* or non *Saccharomyces cerevisiae*) from natural fermentation and study its application in the production process to improve the quality of persimmon wine.

## CONFLICT OF INTEREST

All authors declare that there is no conflict of interest.
